# Targeting Forkhead box O1-aquaporin 5 axis mitigates neuropathic pain in a CCI rat model through inhibiting astrocytic and microglial activation

**DOI:** 10.1080/21655979.2022.2053032

**Published:** 2022-03-24

**Authors:** Yaoping Yu, Meng Wang, Xiao Yu, Yi Yan, Bo Yu, Dayin Zhang

**Affiliations:** a Department of Pain, The First Affiliated Hospital of Nanchang University, Nanchang, China; b Department of Orthopedics, HwaMei Hospital, University of Chinese Academy of Sciences, Ningbo, China

**Keywords:** FoxO1, AQP5, neuropathic pain, inflammation, chronic constriction injury, pathway

## Abstract

Forkhead box O1 (FoxO1) is a critical molecule in modulating cell growth, differentiation and metabolism, acting as a vital transcription factor. This study explored the role of FoxO1 in chronic constriction injury (CCI)-induced neuropathic pain (NP). Microglial and astrocyte activation was achieved with lipopolysaccharide (LPS, 100 ng/mL) to establish an *in-vitro* NP model. Morphological alterations in LPS-induced microglia and astrocytes were assayed by light microscopy. The levels of inflammatory cytokines and proteins in microglia and astrocytes were gauged by enzyme-linked immunosorbent assay (ELISA), and Western blot (WB). The CCI-induced NP rat model was constructed for investigating the FoxO1-AQP5 axis in NP. LPS markedly expanded the expression of inflammatory factors and boosted the expression of FoxO1 and AQP5 in microglia and astrocytes. Inhibition of FoxO1 or AQP5 dramatically decreased the LPS-induced inflammation in microglia and astrocytes. *In vivo*, CCI exacerbated the inflammatory response and NP symptoms and substantially raised the contents of FoxO1 and AQP5 in rats’ spinal cord tissues. Intrathecal administration of the Sirt1 agonist Resveratrol abated CCI-induced activation of FoxO1 and AQP5, abrogated CCI-induced mechanical hyperalgesia and thermal hyperalgesia, depressed microglial and astrocyte activation, and declined the generation of pro-inflammatory mediators in spinal cord tissues. Mechanistically, blocking the FoxO1-AQP5 pathway inactivated the ERK and p38 MAPK pathways. Suppressing the FoxO1-AQP5 axis alleviated CCI-induced NP and inflammatory responses by modulating the ERK and p38 MAPK signaling pathways.

## Graphical abstract

**Figure:**
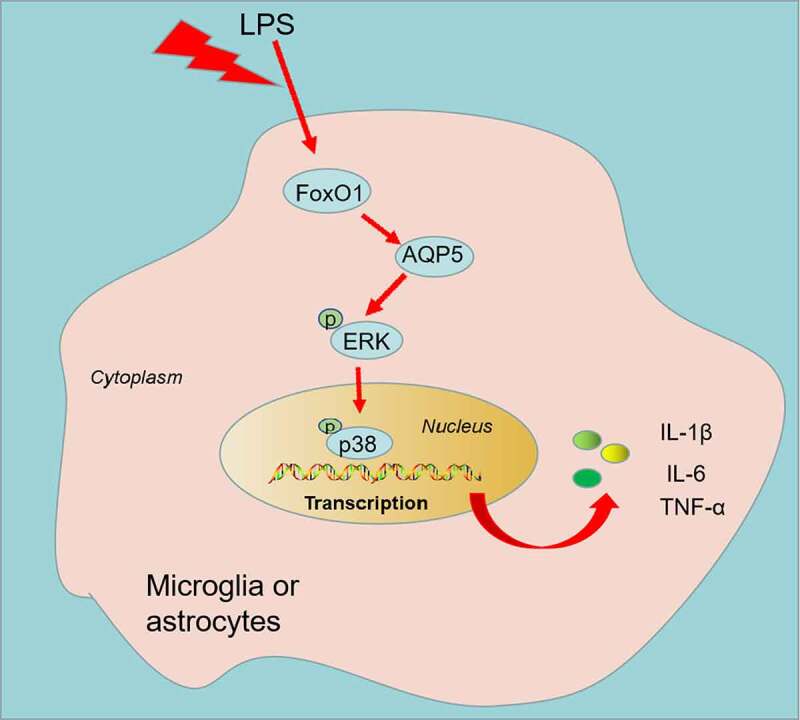

## 1. Introduction

Neuropathic pain (NP) is a chronic secondary pain caused by lesions in the peripheral or central nervous (somatosensory) system [1]. Allodynia (pain elicited by stimuli that do not typically evoke pain) and hyperalgesia (increased pain elicited by stimuli that usually trigger pain) are prominent symptoms in patients with NP [2]. Antidepressants (tricyclics and 5-hydroxytryptamine-norepinephrine reuptake inhibitors) and anticonvulsants (gabapentin and pregabalin) are the first-line drugs for NP. Nevertheless, they only temporarily relieve pain and do not bring long-term improvement [3]. Therefore, understanding the pathogenesis of NP is of great importance in treating NP.

As a conserved transcription factor involved in energy metabolism, forkhead box O1 (FoxO1) contributes to a number of metabolic diseases, such as diabetes, obesity, nonalcoholic fatty liver disease (NAFLD) and atherosclerosis by controlling the transcription of downstream genes that mediate metabolic regulation [4]. As reported, FoxO1 not only exerts a carcinogenic role in cancer [5,6] but also is involved in the evolution of inflammatory diseases. For example, down-regulating FoxO1 dampens the activation of the TLR4/MyD88/MD2-NF-κB inflammatory signaling, chokes mucosal barrier permeability, up-regulates tight junction proteins and amends intestinal mucosal tissue damage in chronic colitis [7]. More importantly, FoxO1 can ameliorate neuroinflammation by regulating the expression of its downstream inflammatory signaling pathways in neuroinflammatory diseases [8]. For instance, suppression of the FoxO1 pathway diminishes LPS-induced neuroinflammation by curbing LPS-induced TLR4 expression and microglial activation [9]. Aquaporin (AQP, comprising 13 members) is an important regulator of the transport of water and small solutes through membranes. Several studies have identified members of the AQP family as being strongly linked to neuroinflammation [^10–13^]. Aquaporin-5 (AQP5), an AQP family member, is located at 12q13.12 and has a length of 1449 bp. According to reports, AQP5 is down-regulated in lung inflammation, and up-regulation of AQP5 mitigates lung inflammation [^14–16^]. FoxO1 and AQP5 expression are strongly interrelated, as FoxO1 directly regulates AQP5 expression in salivary gland alveolar cells via interaction with the AQP5 promoter region [17]. Nonetheless, it is not clear about the role of FoxO1-AQP5 in NP.

Here, we observed that FoxO1-AQP5 was up-regulated in both *in-vivo* and *in-vitro* NP models. Inhibition of FoxO1-AQP5 diminishes inflammatory responses and NP symptoms in chronic constriction injury (CCI) rats and restrains the profiles of p-ERK and p-p38 MAPK in *ex-vivo* and *in-vivo* NP models. Therefore, we hypothesized that suppression of FoxO1-AQP5 may mitigate CCI-induced NP and inflammatory responses by modulating the ERK and p38 MAPK signaling pathways. This study may present a novel option for the treatment of NP.

## 2. Materials and methods

### Cell culture and treatment

Microglia and astrocytes were ordered from the Chinese Academy of Sciences (Shanghai, China). Cells were cultured in the DMEM medium (Thermo Fisher HyClone, Utah, USA) comprising 5% FBS (Thermo Fisher Scientific, MA, USA) and maintained with 5% CO_2_ at 37°C. The medium was substituted every other day, and the cells were sub-cultured every five days. The experiment was carried out when the cells were approximately 90% spread across the bottom of the bottle. Microglia and astrocytes were either treated (M1) or untreated (M0) with lipopolysaccharide (LPS, Sigma-Aldrich, L2880) at 100 ng/mL for 24 hours [18]. The control group was treated with solvent for 24 hours. The morphological alterations in the cells were viewed under an inverted microscope.

### Cell transfection

FoxO1-siRNA (50 nM), AQP5-siRNA (50 nM), and control siRNA (50 nM) were ordered from GenePharma Co., Ltd. (Shanghai, China). Microglia and astrocytes were seeded at 3 × 10^5^ cells/well in a 24-well plate and kept at 37°C with 5% CO_2_ for 24 hours. Then, FoxO1-siRNA, AQP5-siRNA, and control siRNA were transfected into microglia and astrocytes by utilizing Lipofectamine® 3000 (Invitrogen; ThermoFisherScientific, Inc.) following the supplier’s instructions.

### Animals

Male Sprague-Dawley rats (60 rats, acquired from the Animal Experiment Center of Nanchang University) weighing 200–220 g were selected for this study. The rats were placed in standard plastic cages at 24 ± 1°C with 50–70% humidity. All rats were housed individually in cages on a standard 12:12 hour light/dark cycle (lights on from 08:00 to 20:00) and were provided with unlimited water and food. The experimental protocol was endorsed by the Ethics Committee of The First Affiliated Hospital of Nanchang University (Approval number: 2021-8-011), and the study was undertaken as per the regulations published in the NIH Guide for the Care and Use of Laboratory Animals.

### Lentiviral transfection

Recombinant siRNA-FoxO1 (si-FoxO1) and siRNA-NC (si-NC) lentiviral plasmids were acquired from Genepharma (Shanghai, China) with a viral titer of 1 × 10^9^ UT/mL. The model was constructed by intracerebral injection of lentivirus into rats employing a stereotactic instrument, with localization sites as depicted by Li J et al. [19]. The rats were immobilized on the stereotaxic apparatus and their skull was exposed by cutting through the skin of the head. Then, 9 µL of si-NC or si-FoxO1 was injected at a rate of 0.5 µL/minute. All rats were then housed with care for one week in an animal room and tested for FoxO1 mRNA expression before being treated with CCI.

### Chronic constriction injury (CCI) rat model

CCI was utilized to induce the NP rat model. Briefly [20], rats were anesthetized via an intraperitoneal administration of 2% sodium pentobarbital (50 mg/kg). Following skin preparation, an incision of approximately 1 cm is made along the middle of the lower edge of the right femur. The sciatic nerve was revealed by bluntly separating the muscle. The sciatic nerve was ligated at 1 mm intervals with 4.0 wires. The sham group was subjected to the same procedure without ligation. Afterward, the muscle and skin incisions were sutured separately. On the seventh day after CCI, the L4-L5 segments of the rats’ spinal cords were separated, immobilized in 4% paraformaldehyde, dehydrated in gradient ethanol and paraffin-embedded for subsequent testing. Resveratrol (10 μL), a Sirt1 agonist, was injected intrathecally between the L5 and L6 intervertebral spaces using a microinjector and transferred to the cerebrospinal fluid [21]. Rapid tail-flicking of the rats was observed when the needle entered the subarachnoid space.

### Pain threshold assessment

The paw withdrawal threshold (PWT/g) and paw withdrawal latency (PWL/s) of rats in the sham and CCI groups were examined on days 1, 3, 7, 14 and 21, respectively. In simple terms [22], each group of rats was placed in a clear glass box with a metal mesh. The plantar surface of rats was stimulated using von Frey (series 2390; IITC Life Science Inc., Woodland Hills, CA, USA) of logarithmic increasing stiffness (0.4–26 g) until the filaments were slightly bent. The minimum fiber strength that caused rapid paw retraction or withdrawal in rats was recorded. The experiment was replicated 6 times at 5-minute intervals.

PWL was measured in rats using a foot pressure measurement apparatus (No. 37,370; Ugo Basile, Varese, Italy) to estimate thermal hyperalgesia. The rats were kept in a glass box, and the plantar surface of the rat’s left hind paw was stimulated by thermal radiation. The duration between stimulation and paw withdrawal was recorded with a cutoff time of 30 seconds. The experiment was repeated 5 times at 10-minute intervals.

### Enzyme-linked immunosorbent assay (ELISA)

The profiles of inflammatory factors in LPS-induced microglia, astrocytes, and L4-L5 spinal cord tissues of CCI rats were analyzed using ELISA. Supernatants were pooled from spinal cord tissues as described by et al. [23]. Interleukin-1B (IL-1β), interleukin-6 (IL-6), and tumor necrosis factor (TNF-α) ELISA kits (Invitrogen, USA) were applied to examine the concentrations of TNF-α, IL-1β and IL-6 in LPS-induced microglia and astrocytes, as well as in CCI rat spinal cord tissue (L4-L5).

### Western blot (WB)

Protein expression in cells and spinal cord tissues was assayed as per the method of Wu J et al. [24]. Cells and CCI rats’ spinal cord tissues (L4-L5) were harvested and maintained with pre-cooled RIPA lysate (Beyotime Biotcchnology, Shanghai, China) on ice for 20 minutes and centrifuged at 13,000 rpm at 4°C for 20 minutes. The supernatant was taken after centrifugation, and protein quantification was carried out using the BCA Protein Quantification Kit. After that, the protein concentration was adjusted, and the protein was denatured by boiling for 5 minutes. The proteins (50 µg) were then added to 10% SDS-PAGE and transferred to PVDF membranes (Millipore, Bedford, MA, USA). Next, the membranes were sealed with TBST solution comprising 3% TBST and maintained with the diluted antibodies of anti-iNOS (Abcam, MA, USA; 1: 1000, ab178945), anti-COX2 (Abcam, 1: 1000, ab179800), anti-NF-κB (Abcam, 1: 1000, ab32536), anti-p-NF-κB (Abcam, 1: 1000, ab76302), anti-FoxO1 (Abcam, 1:1000, ab219734), anti-AQP5 (Abcam, 1: 1000, ab78486), anti-ERK1/2 (Abcam, 1: 1000, ab184699), anti-p-ERK1/2 (Abcam, 1: 1000, ab223500), anti-p38 MAPK (66,234-1-Ig, Proteintech), anti-p-p38 MAPK (Abcam, 1:1000, ab4822), anti-TLR4 (Abcam, 1:1000, ab13556), anti-MyD88 (Abcam, 1:1000, ab219413), anti-JAK1 (Abcam, 1:1000, ab133666), anti-pJAK1 (Abcam, 1:1000, ab138005), anti-STAT6 (Abcam, 1: 1000, ab32520), anti-pSTAT6 (Abcam, 1: 1000, ab263947), and anti-GAPDH (Abcam, 1: 1000, ab9485) at 4°C overnight. Next, the membranes were subjected to 5 washes of TBST for 3 minutes each. Then, the membranes were spiked with the diluted secondary antibody (1:2000), shaken gently for 40 minutes at room temperature (RT) and rinsed 6 times with TBST (3 minutes each time). At last, the membranes underwent ECL development, fixation and scanning (Beyotime Biotechnology, Shanghai, China). The grayscale values of each band were analyzed using ImageJ, and the ratio of the grayscale value of the target protein to that of GAPDH was adopted as the relative protein expression.

### Immunohistochemistry (IHC)

Spinal cord tissues (L4-L5) were routinely paraffin-embedded, sectioned, dewaxed and hydrated before antigen repair with proteinase K (0.2 mg/mL) and 10 m/Vl sodium citrate solution (pH 6.0). The sections were closed with 2% bovine serum albumin (BSA) for 30 minutes at RT and then kept with rat anti-GFAP monoclonal antibody (1:500; Millipore, USA) and rabbit anti-lbal monoclonal antibody (1:300; Wako Pure Chemical Industries, Ltd Japan), respectively, for 24 hours at 4°C. Next, they were maintained with the HRP-labeled secondary antibody for 2 hours and developed with diaminobiphenyl (DAB) amine solution. The number of GFAP-positive astrocytes and Ibal-positive microglia in spinal cord tissues was estimated by utilizing the Image-Pro Plus image analysis software system (Media Cybernetics, USA) [25].

### Immunofluorescence

The L4-L5 spinal cord of CCI rats was frozen and sectioned (15-uM thick), and then the sections were subjected to IBA1 and GFAP immunofluorescence staining. Tissue sections were excised and blocked with 10% sheep serum for 2 hours at 37°C and then kept overnight at 4°C with a primary antibody of IBA1 (1:100) and GFAP (1:100), respectively. The sections were subjected to three washes in PBS and then left for 2 hours at RT with FITC Anti-mouse 555 (1:500) as secondary antibodies respectively. Next, sections were flushed three times with PBS and DAPI (Sigma-Aldrich, USA) was applied to stain the nuclei. Sections were viewed under a confocal microscope (Olympus, Tokyo, Japan) (200x). Spinal cord tissues from each rat were randomly stained in three tissue sections, with five randomly chosen fields of view photographed in each section [26].

### Data analysis

The results were statistically analyzed with the SPSS 17.0 statistical software. The measurements were presented as ‘mean ± standard deviation’ (x ± s), and all experiments were repeated 3 times. For comparison between the two groups, the Student t-test was applied. One-way analysis of variance (ANOVA) was adopted to compare data between multiple groups. *P* < 0.05 signified statistical significance.

## 3. Results

### LPS activated the FoxO1-AQP5 pathway in microglia and astrocytes

**Figure 1.**
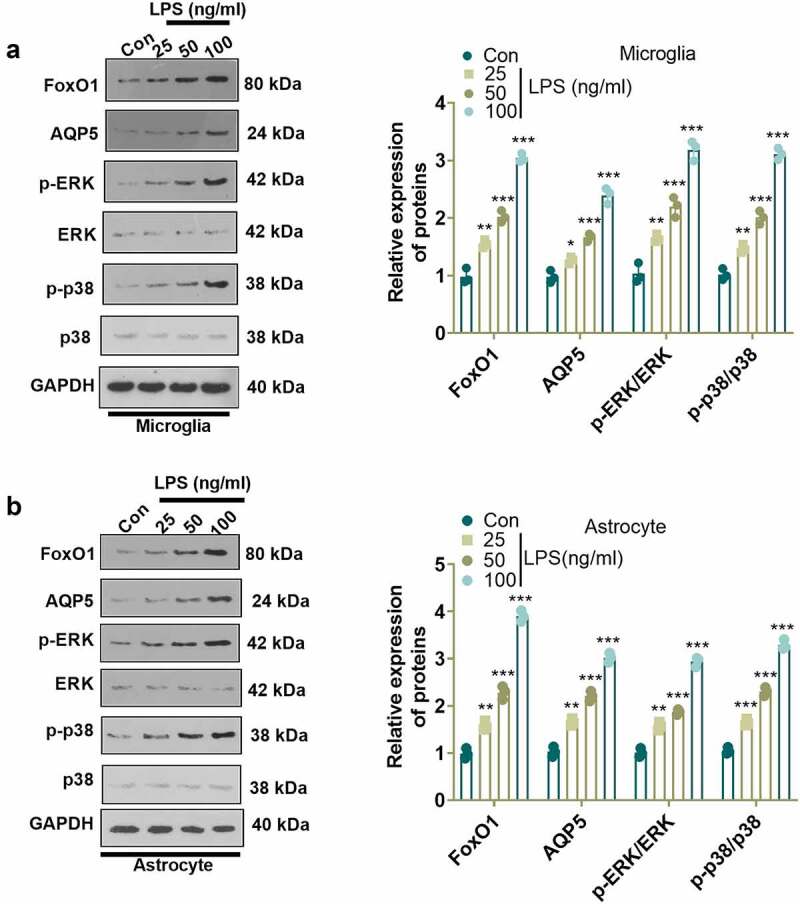
LPS triggered the FoxO1-AQP5 pathway in microglia and astrocytes. (a) and (b) WB examined the expression of FoxO1 and the downstream AQP5 and ERK/p38 MAPK signaling pathways in microglia and astrocytes induced with and without LPS. Data are presented as mean ± SEM (n = 3). **P < 0.01, ***P < 0.001 (vs. Con group).

To check the specific mechanism of LPS in NP, we employed LPS (100 ng/mL) to induce microglia and astrocyte activation, and verified LPS-induced cell activation by light microscopy, ELISA, and WB (Supplementary Figure 1). Previous research has established that FoxO1 contributes to neuroinflammation **[**27**]**. We administered different concentrations of LPS (25, 50, 100 ng/mL) to microglia and astrocytes for 24 hours and examined their influence on the FoxO1, AQP5 and ERK/p38 MAPK pathways in microglia and astrocytes using WB. The results revealed that LPS concentration-dependently augmented the expression of FoxO1 and AQP5 and enhanced the phosphorylation of ERK and p38 MAPK in microglia and astrocytes as compared to the Con group (Figure 1(a,b)). Those data supported that the FoxO1-AQP5 axis plays a role in mediating LPS-induced effects.

### Inhibition of FoxO1 or AQP5 diminished the production of inflammatory factors in LPS-induced cells

**Figure 2.**
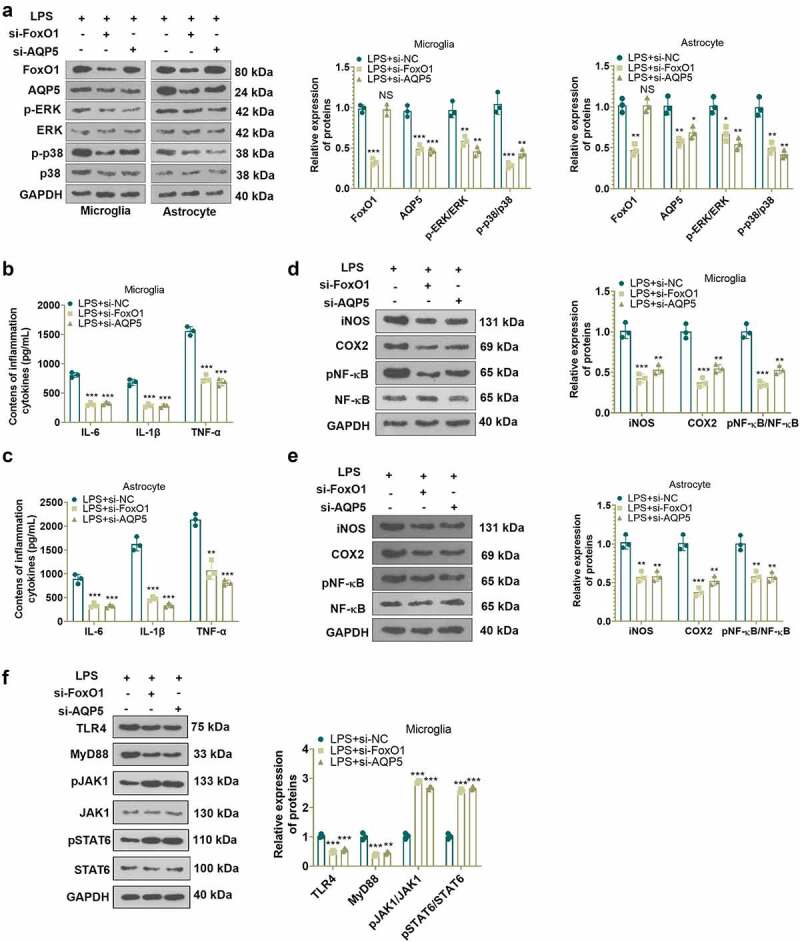
Inhibition of FoxO1 or AQP5 abated LPS-induced production of inflammatory factors in cells.

To test the role of FoxO1 or AQP5 in an *in-vitro* model of NP, we transfected si-FoxO1, si-AQP5 and si-NC into LPS-induced microglia and astrocytes and gauged the profiles of FoxO1 and AQP5 in the cells with WB. The outcomes displayed that inhibition of FoxO1 induced reduced expression of FoxO1 and AQP5 and weakened phosphorylation of ERK and p38 MAPK in microglia and astrocytes versus the LPS+si-NC group. In parallel, compared to the LPS+si-NC group, inhibition of AQP5 led to a declined expression of AQP5 and attenuated phosphorylation of ERK and p38 MAPK in microglia and astrocytes, although there was little change in FoxO1 expression (Figure 2(a)). Besides, ELISA and WB outcomes disclosed that inhibition of FoxO1 or AQP5 substantially declined the contents of IL-6, IL-1β and TNF-α (Figure 2(b,c)), depressed the profiles of iNOS and COX2 and abrogated the phosphorylation of NF-κB in LPS-induced microglia and astrocytes versus the LPS+si-NC group (Figure 2(d,e)). Thus, suppression of FoxO1 or AQP5 decreased the production of inflammatory factors in LPS-treated cells. It was suggested that FoxO1-AQP5 plays a pro-inflammatory role.

si-FoxO1, si-AQP5 and si-NC were transfected into LPS-induced microglia and astrocytes and cultured for 24 hours. A: WB evaluated the profiles of FoxO1, AQP5 and the ERK/p38 MAPK pathway in microglia and astrocytes. B-C: The contents of IL-6, IL-1β and TNF-α in microglia and astrocytes were monitored by ELISA. D-E: WB checked the protein expression of iNOS, COX2, and pNF-κB in microglia and astrocytes. *P < 0.05, **P < 0.01, ***P < 0.001 (vs. LPS+si-NC group).

### CCI rats experienced neuropathic pain accompanied by an accumulation of inflammatory factors in spinal cord tissue

**Figure 3.**
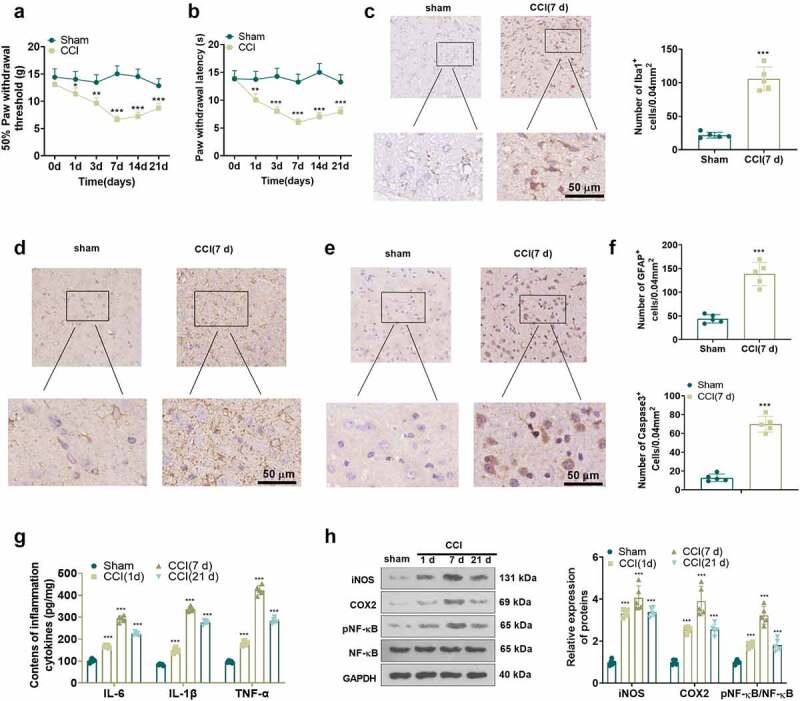
**CCI rats experienced NP, accompanied by increased inflammatory factors in spinal cord tissues**. (a) and (b) PWT and PWL in CCI rats 1, 3, 7, 14 and 21 days after CCI. (c-f) IHC was employed to analyze iBA1-, GFAP-, and Caspase3-positive cells in microglia and astrocytes. (g) The expression of neutron IL-6, IL-1β and TNF-α in spinal cord tissues of CCI rats at 1, 7, and 21 days after operation was gauged by ELISA. (h) The levels of iNOS, COX2 and p-NF-κB in the spinal cord of CCI rats were measured by WB at 1, 7, and 21 days after the operation. Data are expressed as mean ±SEM (n = 5). **P < 0.01, ***P < 0.001 (vs Sham group).

As neuroinflammation contributes to the progression of NP, we induced NP in rats through the construction of the CCI model. As depicted in the figure, PWT and PWL in the CCI group declined from day 1 after CCI versus the Sham group, and the decrease was most pronounced on day 7 and lasted until day 21 (Figure 3(a,b)), implying that CCI caused NP in the rats. It is well known that activation of microglia and astrocytes is a major trigger of NP, and that IBA1 and GFAP are markers of microglia and astrocytes [28]. Accordingly, we assayed the expression of IBA1 and GFAP in spinal cord tissues of CCI rats with IHC. As a result, IBA1-, GFAP- and Caspase3-positive cells were more abundant in the spinal cord tissues of CCI rats (day 7) versus the sham-operated group (Figure 3(c-f)). We measured the levels of IL-6, IL-1β and TNF-α and the inflammatory proteins iNOS, COX2 and p-NF-κB in the spinal cord of CCI rats on 1, 7, and 21 days postoperatively using ELISA and WB. The outcomes displayed uplifted levels of IL-6, IL-1β and TNF-α (Figure 3(g)), up-regulated iNOS and COX2 and augmented phosphorylation of NF-κB (Figure 3(h)) in the spinal cord tissue of CCI rats versus the Sham group. The above findings hinted that inflammation plays an important role in the CCI rats.

### FoxO1-AQP5 was up-regulated in the spinal cord of CCI rats

**Figure 4.**
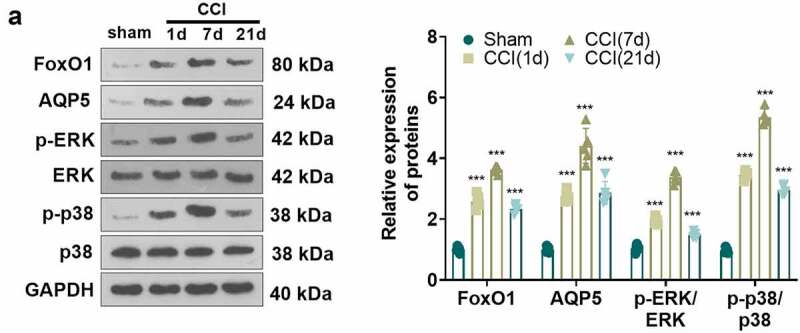
**FoxO1-AQP5 was up-regulated in spinal cord tissues of CCI rats**. (a) WB assayed the expression of FoxO1, AQP5, p-ERK and p-p38 in the spinal cord tissue of CCI rats at 1, 7, and 21 days postoperatively. Data are expressed as mean ± SEM (n = 5). ***P < 0.001 (vs. Sham group).

To test the contribution of the FoxO1-AQP5 pathway in an *in-vivo* model of NP, we measured the protein expression of FoxO1 and AQP5 in the spinal cord of CCI rats at 1, 7, and 21 days postoperatively using WB. Notably, compared to the Sham group, CCI rats exhibited up-regulation of FoxO1 and AQP5 and potentiated phosphorylation of ERK and p38 MAPK in spinal cord tissues, peaking at day 7 (Figure 4(a)). The above results indicated that FoxO1-AQP5 expression was elevated in the spinal cord tissue of CCI rats, which might affect the evolution of NP.

### Inhibition of FoxO1 dampened neuropathic pain and production of inflammatory factors in spinal cord tissues of CCI rats

**Figure 5.**
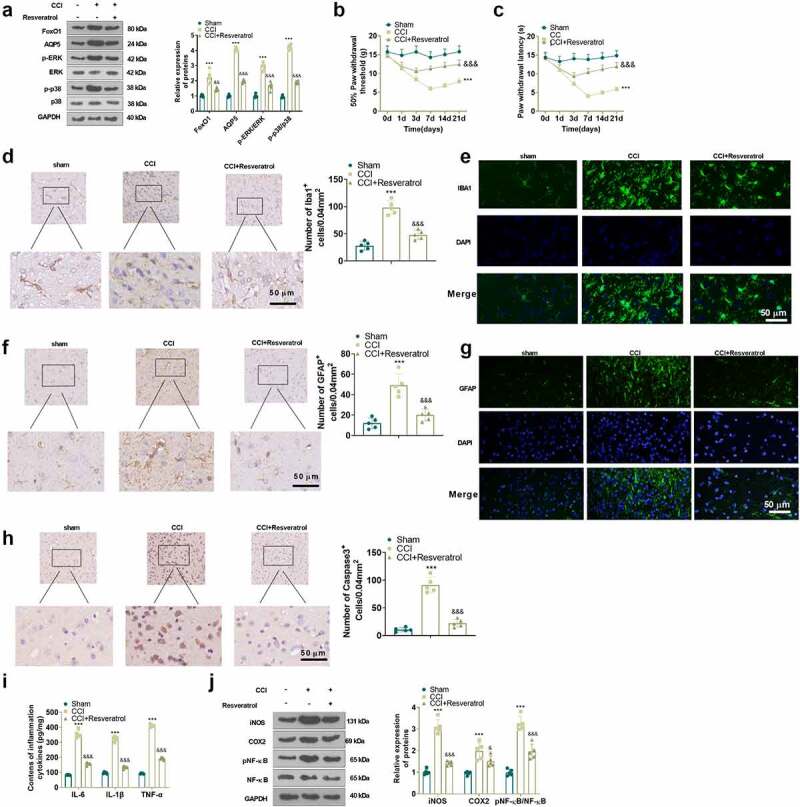
Inhibition of FoxO1 attenuated nerve pain and inflammatory cytokine production in the spinal cord in CCI rats.

**Figure 6.**
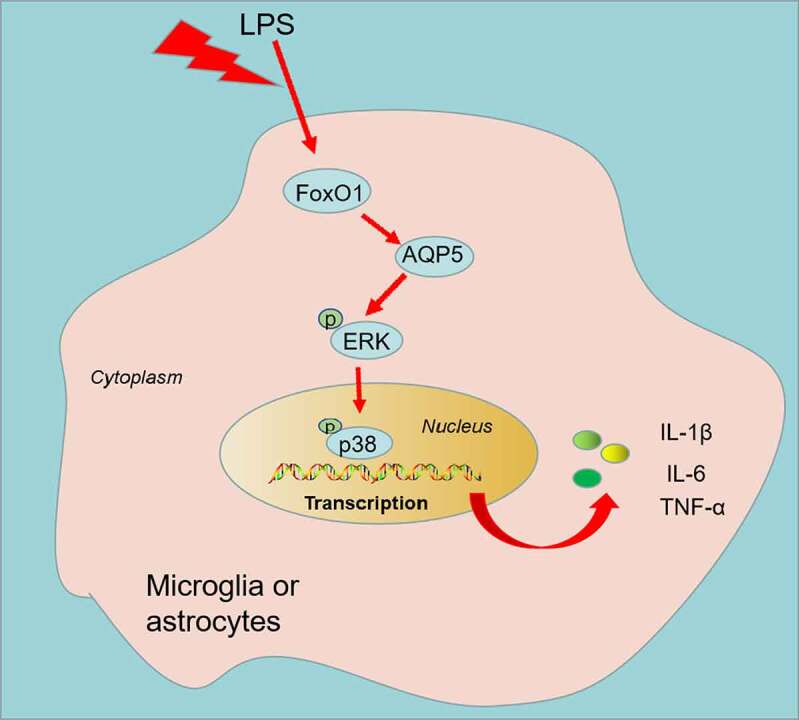
The mechanism’s diagram.

To characterize the function of FoxO1 in CCI rats, we administered the Sirt1 agonist Resveratrol (10 μL) intrathecally into the brain of CCI rats via a microinjector on the first day following CCI surgery. The expression of FoxO1 and AQP5 in the spinal cord of CCI rats was gauged using WB on day 3 after injection. The findings presented that Resveratrol treatment curbed the expression of FoxO1 and AQP5 and restrained the phosphorylation of ERK and p38 MAPK in the spinal cord tissue of CCI rats versus the CCI group (Figure 5(a)). Additionally, Resveratrol administration resulted in facilitation in PWT and PWL (Figure 5(b,c)) in CCI rats versus the CCI group. IHC and immunofluorescence displayed that Resveratrol treatment diminished apoptosis of IBA1-, GFAP- and Caspase3-positive cells and neurons (Figure 5(d-h)). As exhibited by ELISA and WB, in contrast to the CCI group, Resveratrol markedly restricted the levels of IL-6, IL-1β and TNF-α (Figure 5(i)), depressed the protein expression of iNOS and COX2, and damped the phosphorylation of NF-κB in the spinal cord of CCI rats (Figure 5(j)). Thus, inhibition of FoxO1 abated NP and production of inflammatory factors in spinal cord tissues in CCI rats (Figure 6).

Resveratrol (10 μL), a Sirt1 agonist, was delivered intrathecally via microinjector into the brains of CCI rats on the first day after CCI surgery. (a) The protein profiles of FoxO1, AQP5, p-ERK and p-p38 in spinal cord tissues of CCI rats were measured with WB on the third day after CCI surgery. (b) and (c) PWT and PWL of CCI rats at 1, 3, 7, 14 and 21 days after CCI surgery. (d)-(h) IHC or immunofluorescence were performed to determine apoptosis of IBA1, GFAP in the spinal cord. Caspase3-positive cells and neurons in CCI rats on the third day after CCI. Scale bar = 50 μm. (i) The levels of IL-6, IL-1β and TNF-α in spinal cord tissues of CCI rats were analyzed by ELISA on the third day after surgery. (j) The profiles of iNOS, COX2, and p-NF-κB in spinal cord tissues of CCI rats were determined by WB. *P < 0.05, ***P < 0.001 (vs. Sham group), &&&&*P* < 0.001 (vs. CCI group).

FoxO1-AQP5 axis becomes upregulated in LPS-activated microglia and astrocyte, and enhances inflammatory cytokines expression.

## 4. Discussion

NP is a result of a lesion or disease influencing the somatosensory system with a prevalence of 6.9–10% in the population [29]. Recent research has highlighted the important role of astrocytes and microglia in central sensitization and chronic pain [30,31]. Specifically, microglia and astrocytes are activated following nerve injury, and these activated glial cells release cytokines such as IL-6, IL-1β, and TNF-α, leading to NP [32,33]. Blocking the activation of microglia and astrocytes can effectively alleviate the NP symptoms induced by nerve injury [34,35]. In this study, we modeled the CCI by ligating the sciatic nerve, with a reduction in PWT and PWL indicating successful modeling. Inhibition of the FoxO1-AQP5 axis expression was effective in relieving CCI-induced NP and inflammatory responses in rats, possibly through modulation of the ERK and p38 MAPK pathways.

FoxO1 is highly expressed in neurons of various sympathetic ganglia and is a key transcription factor in DA neurons. FoxO1 is responsible not only for the modulation of energy and glucose/insulin homeostasis but also for TH expression and sympathetic nerve activity [36]. Numerous investigations have documented that inhibition of FoxO1 diminishes microglial inflammation [27,37]. More importantly, FoxO1 can be applied as a biomarker for predicting and diagnosing chronic pain. FoxO1 knockdown minimizes rats’ sensitivity to static mechanical stimuli and mitigates static mechanical abnormalities in a rat model of neuropathic, inflammatory and chemotherapeutic pain [38]. Here, we identified that FoxO1 was up-regulated in LPS-induced microglia or astrocytes and in CCI rats’ spinal cord tissues and that inhibition of FoxO1 expression suppressed the inflammatory response and NP in CCI rats. Besides, microglia are known to mediate multiple aspects of neuroinflammation and play a dual role in various brain diseases through different microglia phenotypes (the harmful M1 phenotype and the neuroprotective M2 phenotype). Therefore, the conversion of microglia from type M1 to type M2 by modulation is a potential therapeutic strategy for neuroinflammatory disorders [39]. In parallel, the TLR4/MyD88/NF-κB pathway and the JAK1/STAT6 signaling pathway are essential in modulating M1/2 polarization in microglia [^40–42^]. Here, we further elicited the specific mechanisms by which FoxO1 affects LPS-induced phenotypic transition in microglia. The outcome suggests that suppression of FoxO1 activates the JAK1-STAT6 pathway by dampening the TLR4/MyD88/NF-κB axis, thereby fostering the conversion of microglia from M1 to M2 phenotype. These outcomes imply that FoxO1 may be an important referential intervention target for treating NP in humans, which, however, also needs to be validated in CCI patients.

Aquaporins are a family of membrane aquaporins. There are 13 known aquaporins in mammals, whose main function is to regulate intracellular and intercellular water flow [43]. Earlier research has established that AQP1, AQP4, AQP2 and AQP9 are associated with spinal cord injury- or nerve injury-induced nerve pain in rats [^44–47^]. AQP5, an AQP family member, is highly expressed in astrocytes and is related to the hypoxia of astrocytes [48]. Nevertheless, the action of AQP5 in CCI-induced NP is poorly documented. Extracellular signal-regulated kinases (ERK) 1 and 2 belong to the mitogen-activated protein kin (MAPK) family. As reported, peripheral nerve injury triggers ERK activation in glial cells, sensory neurons and secondary neurons, and inhibition of ERK curbs NP induced by peripheral nerve injury [49]. For example, the ERK/p38 pathway in the dorsal root ganglion (DRG) is motivated in a spinal nerve ligation (SNL)-induced NP rat model, and inhibition of p38 activation lessens SNL-induced thermal hyperalgesia [50], as disclosed in research by Jin SX, et al. [51]. On the other hand, FoxO1 can target and regulate AQP5 [13], which modulates the profiles of p-ERK and p-p38 MAPK [52]. Nevertheless, the role of FoxO1, AQP5, and the ERK/p38 MAPK pathway in NP is unclear. Here, we discovered that FoxO1, AQP5 and ERK/p38 MAPK signaling pathways were significantly up-regulated in LPS-induced microglia or astrocytes as well as in CCI rats’ spinal cord tissues. Meanwhile, inhibition of FoxO1-AQP5 expression restrained phosphorylation of ERK and p38 MAPK, reducing inflammatory responses and NP in CCI rats. Hence, we posited that the inhibitory effect of FoxO1 on neuroinflammation might be achieved through its inhibition on AQP5 and the activity of the ERK/p38 MAPK pathway.

## Conclusion

Overall, we demonstrated that inhibition of FoxO1-AQP5 suppressed LPS-induced inflammatory responses in microglia and astrocytes and hampered neuronal apoptosis and NP in CCI rats by blocking the ERK/p38 MAPK pathway. These outcomes may bring a novel therapeutic target for treating NP. Nevertheless, the role of FoxO1 in NP requires further study to fully elucidate the association of FoxO1 with the development of NP.

## Supplementary Material

Supplemental Material

## Data Availability

The data sets used and analyzed during the current study are available from the corresponding author on reasonable request.

## Abbreviations

AQP5 aquaporin 5

CCI chronic constriction injury

COX-2 cyclooxygenase-2

DAPI 4ʹ6-diamidino-2-phenylindole

DMSO dimethyl sulfoxide

ERK extracellular signal-regulated kinase

FoxO1 Forkhead box O1

FBS Fetal bovine serum

iNSO Inducible nitric oxide synthase

IL-1β interleukin-1beta

IL-6 Interleukin 6

IHC Immunohistochemistry

JAK1 Janus kinase 1

LPS lipopolysaccharide

MyD88 Myeloid differentiation primary response gene 88

NS No significance

NP Neuropathic pain

NFκB The nuclear transcription factor-kappa B

P38 mitogen-activated protein kinase

qRT-PCR quantitative real-time polymerase chain reaction

si-NC Small interfering RNA negative control

STAT6 Signal transducer and activator of transcription 6

TLR4 toll-like receptor-4

TNF-α Tumour Necrosis Factor alpha

WB Western blot
