# Supplementary figures and images for: Targeting Forkhead box O1-aquaporin 5 axis mitigates neuropathic pain in a CCI rat model through inhibiting astrocytic and microglial activation
Source: Bioengineered. 2022 Mar 24;13(4):8567–80. doi: 10.1080/21655979.2022.2053032 (PMC9161847; doi:10.1080/21655979.2022.2053032)

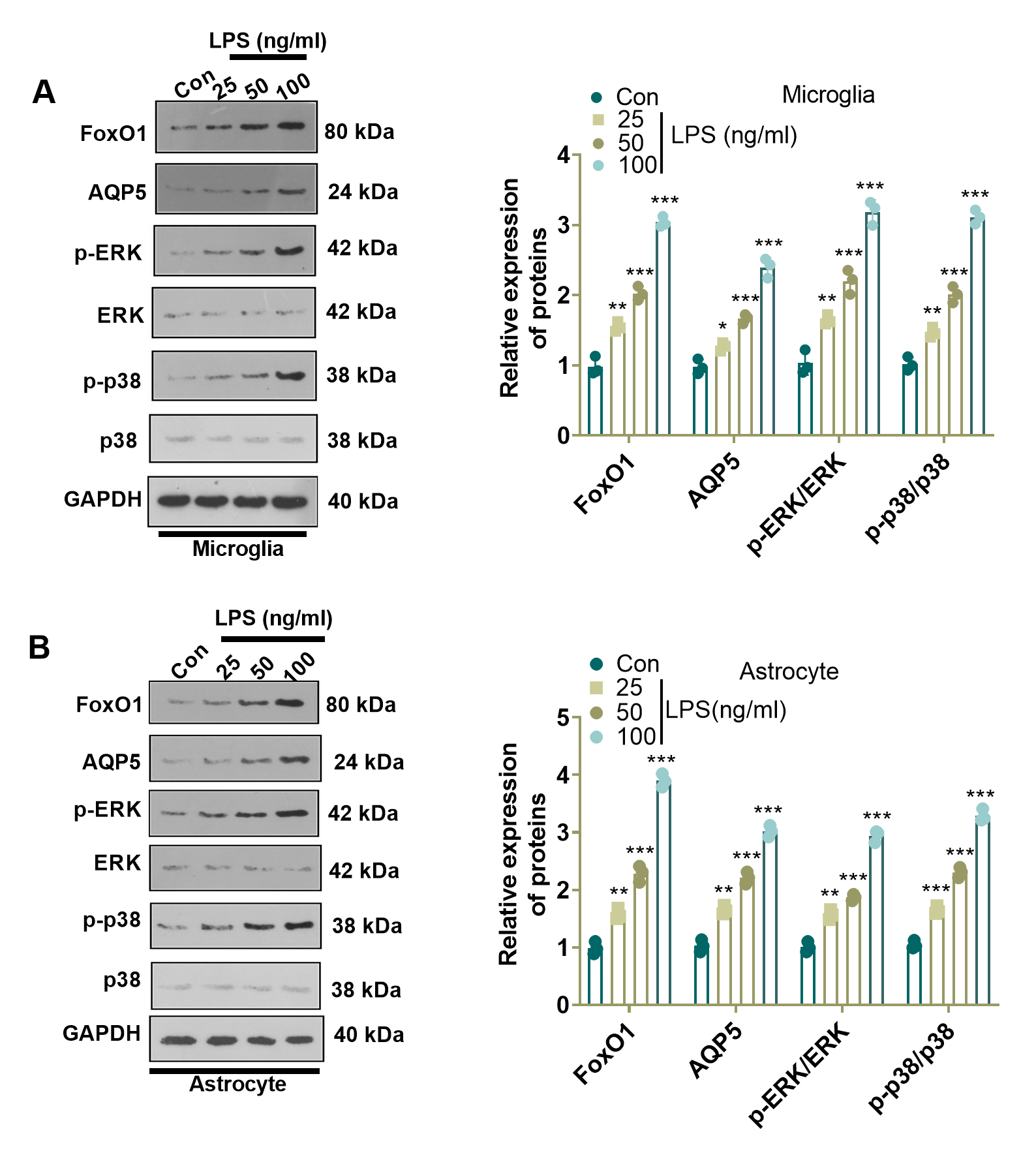

Supplement: Supplemental Material [file KBIE_A_2053032_SM7177.zip › Supplementary Figure 1.tif]

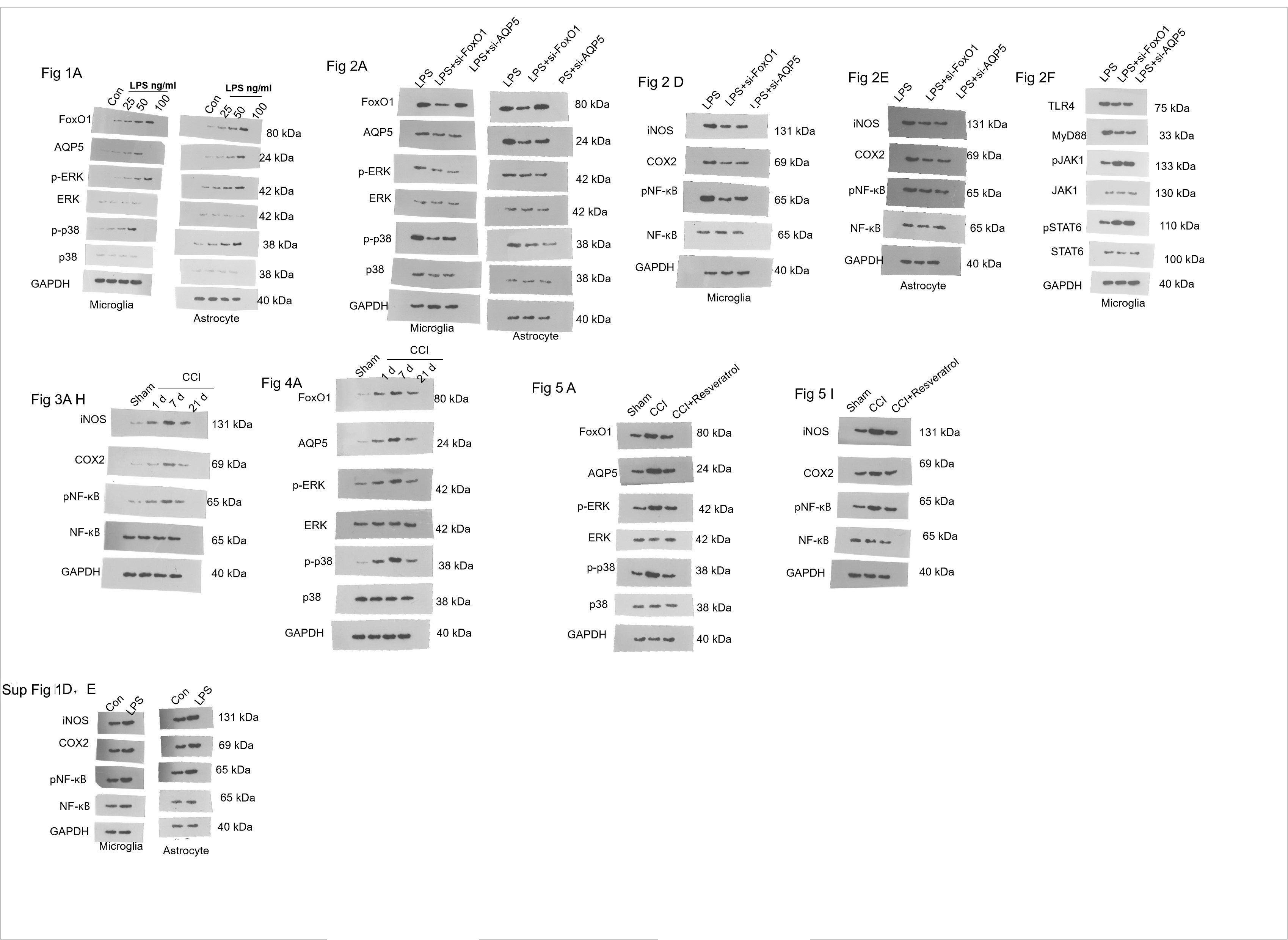

Supplement: Supplemental Material [file KBIE_A_2053032_SM7177.zip › supplementary figure2.jpg]
